# Down-regulation of *RBP4* indicates a poor prognosis and correlates with immune cell infiltration in hepatocellular carcinoma

**DOI:** 10.1042/BSR20210328

**Published:** 2021-04-16

**Authors:** Mingxing Li, Zhihui Wang, Lixu Zhu, Yifang Shui, Shuijun Zhang, Wenzhi Guo

**Affiliations:** 1 Department of Hepatobiliary and Pancreatic Surgery, The First Affiliated Hospital of Zhengzhou University, Zhengzhou 450052, China; 2 Key Laboratory of Hepatobiliary and Pancreatic Surgery and Digestive Organ Transplantation of Henan Province, The First Affiliated Hospital of Zhengzhou University, Zhengzhou 450052, China; 3 Open and Key Laboratory of Hepatobiliary and Pancreatic Surgery and Digestive Organ Transplantation at Henan Universities, Zhengzhou 450052, China; 4 Henan Key Laboratory of Digestive Organ Transplantation, Zhengzhou 450052, China

**Keywords:** hepatocellular carcinoma, prognosis, RBP4

## Abstract

Recent research has indicated that metabolically related genes play crucial roles in the pathogenesis of hepatocellular carcinoma (HCC). We evaluated the associations between novel biomarkers and retinol-binding protein 4 (RBP4) for predicting clinical HCC outcomes, hub-related genes, pathway regulation, and immune cells infiltration. Bioinformatic analyses based on data from The Cancer Genome Atlas were performed using online analysis tools. RBP4 expression was low in HCC and was also down-regulated in pan-cancers compared with normal tissues. RBP4 expression was also significantly different based on age (41–60 years old versus 61–80 years old), and low RBP4 expression levels were associated with advanced tumor stages and grades. Higher RBP4 expression was associated with better overall survival time in HCC patients, and we identified a deletion-mutation rate of 1.4% in RBP4. We also identified ten co-expressed genes most related to RBP4 and explored the relationships between six hub genes (*APOB, FGA, FGG, SERPINC1, APOA1*, and *F2*) involved in RBP4 regulation. A pathway enrichment analysis for RBP4 indicated complement and coagulation cascades, metabolic pathways, antibiotic biosynthesis pathways, peroxisome proliferator-activated receptor signaling pathways, and pyruvate metabolism pathways. These results suggest that RBP4 may be a novel biomarker for HCC prognosis, and an indicator of low immune response to the disease.

## Introduction

Hepatocellular carcinoma (HCC) can occur after years of chronic liver disease and comprises more than 90% of primary liver cancers. HCC is the second leading cause of cancer-related deaths worldwide and is responsible for nearly 800,000 deaths annually [1,2]. Associated problems with HCC include early-diagnosis inaccuracies and a lack of clarity for optimal methods to improve treatment, to monitor disease progression, and to establish recurrence. Therefore, an accurate marker for early detection and prediction of clinical survival and therapeutic responses would be valuable for the clinical management of HCC.

Carcinogen-mediated molecular mechanisms are not only involved in the genesis of cancer but also metabolically in the tumor-associated microenvironment [3]. Metabolic disorders are known to increase the risk of cancer and cancer-related mortality [4]. Metabolic alterations in tumor-microenvironments related to hyperinsulinemia and obesity are associated with an increased risk for, incidence of, and mortality from breast cancer [5], gastrointestinal cancer [6], lung cancer [7], prostate cancer [8], colorectal cancer [9], and pancreatic cancer [10]. Accumulating evidence has revealed that metabolic disorders can also drive HCC and are associated with its progression [11]. Therefore, a better understanding of metabolism-related molecular mechanisms associated with HCC may lead to detection markers and to potential therapeutic targets.

Retinol binding protein 4 (RBP4) is a 21-kDa protein belonging to the lipocalin family and is a retinol transporter in the blood [12,13]. RBP4 is mainly synthesized and released in the liver and fat, acting as an adipokine with crucial roles in growth, vision, and metabolic diseases [14–16]. RBP4-related disruption of retinol metabolism is also involved in malignancy [17]. Recently, RBP4 has been reported to act as a bridge between obesity and cancer cell metabolism, and serum RBP4 levels in patients with HPA breast cancer have been reported to be significantly higher than those in control patients [18]. RBP4, as an oncogene, has also been implicated in ovarian cancer as one of the elements regulating the Rock1 pathway [19–21]. Studies have also demonstrated that RBP4 activates STRA6, which drives and mediates tumor initiation, tumor growth, and the expression of stemness markers in the genesis of colon cancer [22]. In pancreatic cancer, higher expression levels of RBP4 have also been reported compared with those in normal control subjects [23]. The progression of malignant melanoma has also been linked to a positive association between the *RBP4* gene and hypermethylation [24]. RBP4 may also act as a molecular bridge between the dysregulation of metabolic processes and cancers. Studies have indicated that high serum RBP4 in HCC combined with metabolic syndrome patients were closely associated with poor prognosis [25]. A comparative assessment of RBP4 expression, clinicopathological features, and HCC-patient outcomes has also indicated that RBP4 may be a contributing factor linked to the metabolic microenvironment of HCC [26,27].

In the present study, we performed bioinformatic analyses to explore any relationships between RBP4 expression and HCC patient survival using data from tumor and normal tissue samples. We explored differentially expressed genes, co-expressed genes, hub genes, and immune cell infiltration related to RBP4 in HCC. These explorations may provide novel insights into RBP4 associated function role in HCC initiation, progression, prognosis. HCC prognosis prediction.

## Materials and methods

### Patient data and UALCAN analyses

All the data used in this study (from 371 HCC tumors paired with 50 normal tissue samples) were obtained from The Cancer Genome Atlas (TCGA). UALCAN [28] (http://ualcan.path.uab.edu), an online resource, was used for in-depth analyses of gene expression data using TCGA-Liver Hepatocellular Carcinoma (TCGA-LIHC) datasets. Student’s *t*-tests were applied, and we considered *P*-values<0.05 to be significant.

### RBP4 protein-expression data

We used RBP4 protein level data from the tissue microarrays represented in the Human Protein Atlas [29] (HPA, https://www.proteinatlas.org/). Data from two patients (IDs: 1846 and 2556) were analyzed for the expression of RBP4.

### Correlations between RBP4 expression and clinicopathological characteristics

Possible correlations between RBP4 expression levels and clinicopathological features were also assessed. Clinical prognostic features, such as overall survival (OS), recurrence-free survival (RFS), disease-specific survival (DSS), and progression-free survival (PFS) were compared with RBP4 expression levels, and the results were displayed using Kaplan−Meier plots [30] (https://kmplot.com/).

### Mutation frequencies and co-expressed genes

The online CBioPortal site (https://www.cbioportal.org/) and LinkedOmics [31] were used to access data for *RBP4* gene mutations, co-expressed genes, and mutations related to protein expression. Genomic profiles included mutations, putative copy number alterations from TCGA-LIHC data, and mRNA expression Z-scores (RNA Seq V2 RSEM) using a *Z*-score threshold setting of ± 2.0. In addition, we determined the top 10 genes co-expressed with *RBP4* using Spearman's rank-order correlation, with *R* > 0.7.

### RBP4 pathway-enrichment analyses

Gene ontology (GO) enrichment analyses, Kyoto Encyclopedia of Genes and Genomes (KEGG) pathway enrichment analyses, and protein–protein interaction (PPI) networks were visualized using Network Analyst 3.0.

### RBP4-related immune cell infiltration

We used the Tumor IMmune Estimation Resource (TIMER, https://cistrome.shinyapps.io/timer/), a comprehensive platform for the statistical analyses of immune cell purity and infiltration of B cells, CD4+ T cells, CD8+ T cells, neutrophils, macrophages, and dendritic cells into tumors, to assess RBP4-related immune cell infiltration. Additionally, we explored the expression of RBP4 across pan-cancer through TIMER.

## Results

### RBP4 expression levels in liver cancer

**Figure 1:**
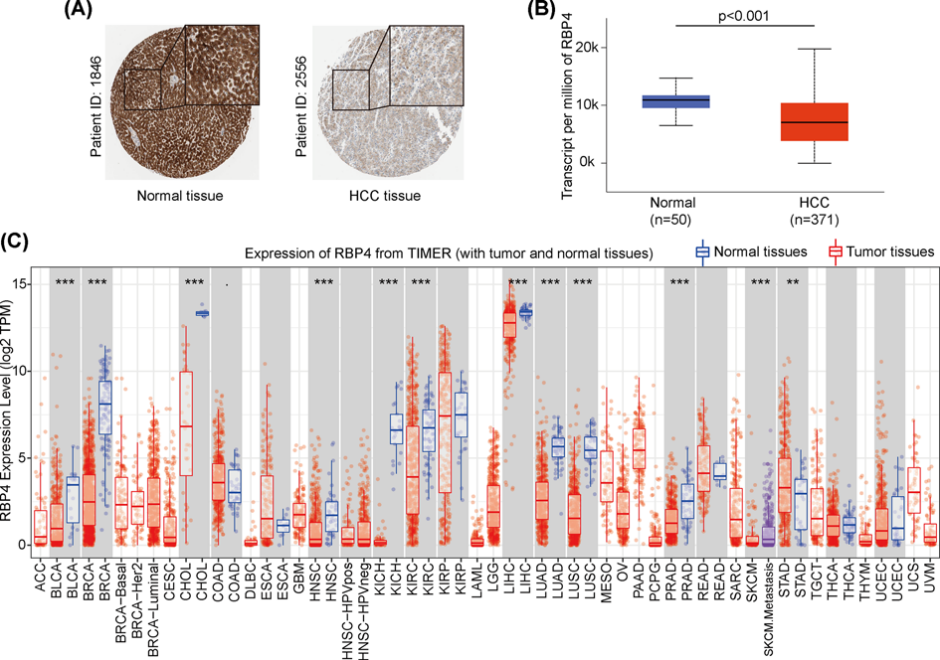
RBP4 expression was lower in hepatocellular carcinoma (HCC) and in various cancers (**A**) RBP4 protein was decreased expressed in HCC tissue. RBP4 was overexpressed in normal liver tissues. (**B**) The level of RBP4 mRNA transcription was lower in HCC samples compared to normal liver samples. (**C**) Pan-cancer analysis of RBP4 mRNA transcription levels showed a lower RBP4 expression in most tumor tissues. **P<0.01, and ***P<0.01

For RBP4 expression levels in HCC and its corresponding normal tissues, we used protein expression data from the HPA database. Using immunohistochemical data, we found that RBP4 protein expression was lower in HCC tissue, but was highly expressed (protein and mRNA) in normal tissue (*P*<0.001, Figure 1A,B). For the pan-cancers comparisons, we utilized TIMER to explored RBP4 expression, and the results showed that RBP4 was highly expressed in most normal tissues compared with its corresponding tumor tissues, including bladder urothelial carcinoma (BLCA), breast invasive carcinoma (BRCA), cholangiocarcinoma (CHOL), head and neck squamous cell carcinoma (HNSC), kidney chromophobe (KICH), kidney renal clear cell carcinoma (KIRC), liver hepatocellular carcinoma (LIHC), lung adenocarcinoma (LUAD), lung squamous cell carcinoma (LUSC), prostate adenocarcinoma (PRAD), skin cutaneous melanoma (SKCM), and stomach adenocarcinoma (STAD). However, RBP4 expression was increased in tumor tissues in STAD, and the differences were statistically significant (Figure 1C). Taken together, these results suggest that RBP4 may be a novel biomarker for HCC diagnosis.

### RBP4-related clinical features and prognoses

**Figure 2:**
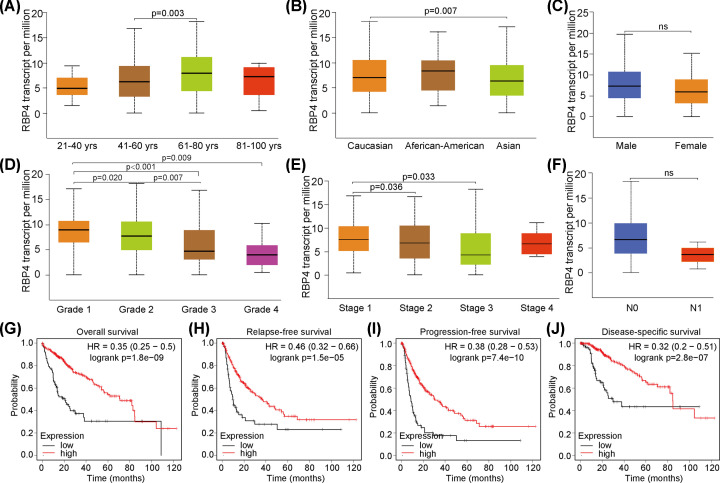
Relationships between RBP4 expression, clinical characteristics, and clinical outcomes (**A**) RBP4 expression levels between different age groups. (**B**) Comparison of RBP4 transcription levels between different ethnic groups. (**C**) Comparison of RBP4 transcription levels based on gender. (**D**) Comparison of RBP4 transcription levels based on tumor grading. (**E**) Comparison of RBP4 transcription levels based on cancer staging. (**F**) Comparison of RBP4 transcription levels based on lymph node metastasis status. (**G**) Overall patient survival according to RBP4 expression levels. High RBP4 expression was associated with a better prognosis. (**H**) Relapse-free survival, according to RBP4 expression, indicated that high expression in HCC was associated with a better prognosis (HR = 0.46, 95% CI: 0.32–0.66), log rank *P* = 1.5e-05). (**I**) Progression-free survival, according to RBP4 expression, indicated that high expression was associated with longer survival (HR = 0.38, 95%CI: 0.28–0.53), log rank *P* = 7.4e-10). (**J**) Disease-specific survival analysis indicated that higher RBP4 expression levels had longer survival time (HR = 0.32, 95% CI: 0.2–0.51), log rank *P* = 2.8e-07).

To further explore the relationship between RBP4 expression and clinical information, we applied the HCC tumor tissues transcriptional profiles, and examined possible subgroup associations with RBP4 expression based on patient age, gender, race, tumor grade, TNM staging, and lymph node metastasis. The results demonstrated that RBP4 expression in the younger group (41–60 years of age) was lower than that in the elder group (61–80 years of age) (*P*=0.003, Figure 2A), and Caucasians had higher RBP4 expression compared to Asians (*P*=0.007, Figure 2B). However, there was no significant difference in RBP4 expression between male and female (Figure 2C). In addition, RBP4 expression levels were higher in advanced tumors (by grading and staging) compared with those in less-advanced tumors, and this difference was significant (Figure 2D,E). However, there was no significance between lymph node metastasis subgroup and no lymph node metastasis subgroup (Figure 2F).

To investigate prognoses for clinical survival, the OS analyses showed that RBP4 overexpression was associated with longer survival time (hazard ratio (HR) = 0.35, 95% confidence interval (95% CI): 0.25–0.5, (log rank *P* = 1.8e-0, Figure 2G). The analysis of RFS indicated that higher RBP4 expression levels were also associated with longer RFS time (HR = 0.46, 95% CI: 0.32–0.66), (log rank *P* = 1.5e-05, Figure 2H), and the PFS analysis confirmed that up-regulated RBP4 had longer PFS time (HR = 0.38, 95% CI: 0.28–0.53), (log rank *P* = 7.4e-10, Figure 2I). For DSS, high RBP4 expression was also associated with better outcomes (HR = 0.32, 95% CI: 0.2–0.51), (log rank *P* = 2.8e-07, Figure 2J). Overall, these results indicate that high RBP4 expression was an indicator for improved prognoses in patients with HCC, suggesting that elevated RBP4 expression may represent a good prognosis indicator for HCC progression.

### RBP4-related gene mutations and immune cell infiltration characteristics

**Figure 3:**
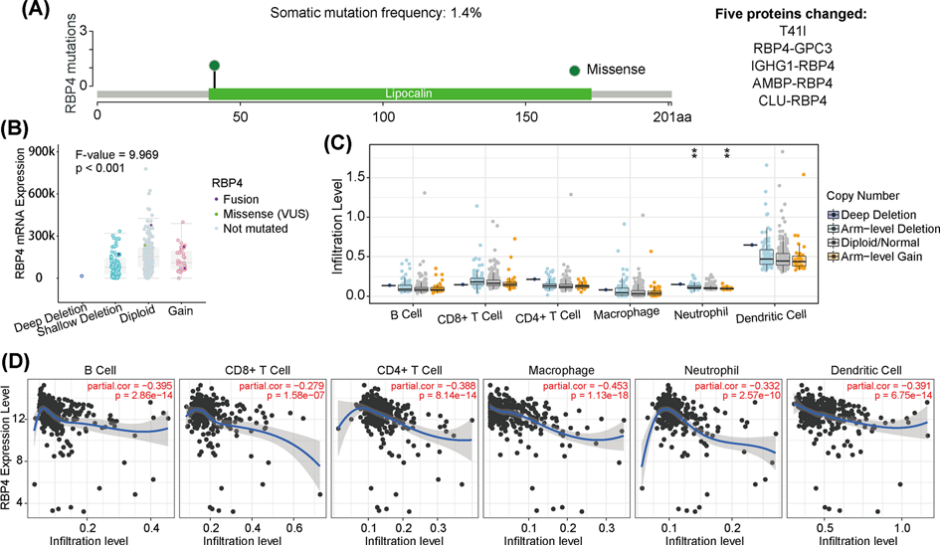
RBP4 mutation types, related protein expressions, and immune cell infiltration (**A**) Deletion-type mutations made up 1.4% of total *RBP4* gene mutations, with five protein changes: T41l, RBP4-GPC3, IGHG1-RBP4, AMBP-RBP4, and CLU-RBP4. (**B**) *RBP4* mutations affected RBP4 mRNA expression levels. (**C**) Different types of *RBP4* mutations also affected immune cell infiltration levels, with neutrophils showing significant differences. (**D**) Analysis of immune cell purity, and the relationship between different immune cells and RBP4 expression levels.

Deletion mutations accounted for 1.4% of total RBP4 mutations, and five proteins were changed because of RBP4 related somatic mutations (T41l, RBP4-GPC3, IGHG1-RBP4, AMBP-RBP4, and CLU-RBP4) (Figure 3A). The different types of RBP4 mutations also affected RBP4 m RNA expression (*F*-value = 9.969, *P*<0.001, Figure 3B). The RBP4 mutation types were also correlated with immune cell infiltration levels, especially for neutrophils with deep-deletion and arm-level gain types of mutations (Figure 3C). RBP4 expression was also linked to immune cell purity, with B cells, CD8+ T cells, CD4+ T cells, macrophages, neutrophils, and dendritic cells all negatively correlated with RBP4 expression (Figure 3D). Collectively, RBP4 expression and mutations were associated with immune cell infiltration characteristics.

### Differentially expressed genes associated with RBP4

**Figure 4:**
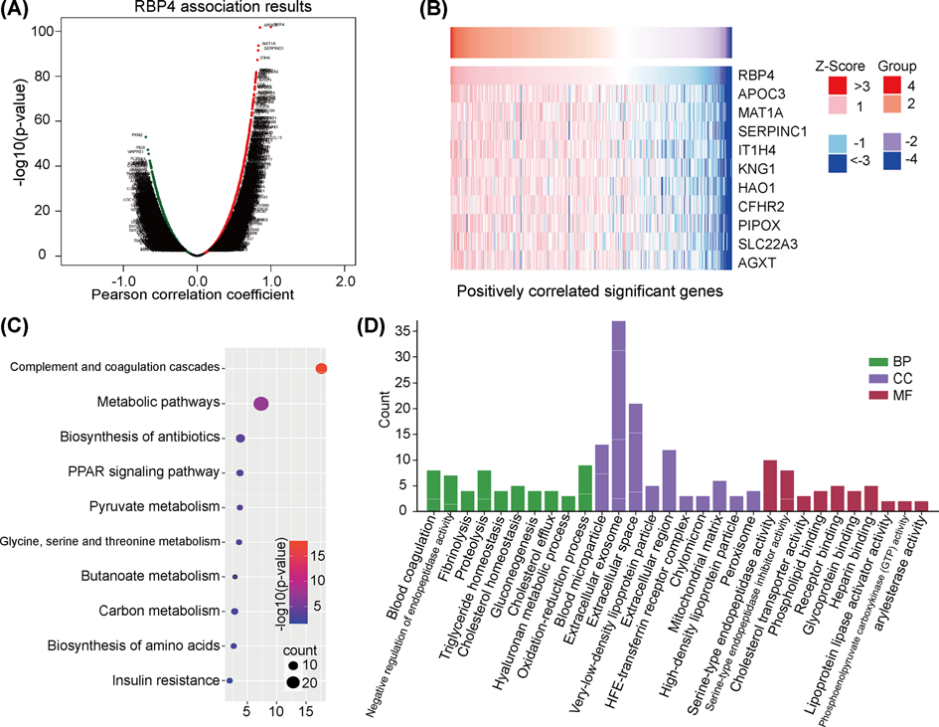
Differentially expressed genes related to RBP4 (**A**) A volcano plot showing significant positively and negatively correlated genes related to RBP4 expression. (**B**) A heat map of the top 10 positively correlated genes related to RBP4 expression in HCC. (**C**) A KEGG enrichment analysis of pathways, including complement and coagulation cascades, metabolic pathways, the biosynthesis of antibiotics, the peroxisome proliferator-activated receptor signaling pathway, and pyruvate metabolism. (**D**) RBP4-related pathway enrichment analyses. Biological processes (BP): blood coagulation; negative regulation of endopeptidase activity, fibrinolysis, proteolysis, triglyceride homeostasis, and cholesterol homeostasis. Cellular components (CC): blood microparticles, extracellular exosomes, the extracellular space, very low-density lipoprotein particles, and the extracellular region. Molecular functions (MF): serine-type endopeptidase activity, serine-type endopeptidase inhibitor activity, cholesterol transporter activity, phospholipid binding, receptor binding, glycoprotein binding, and heparin binding.

To explore the relationship between *RBP4* gene expression and other differentially expressed genes, we examined the positive and negative correlations for other gene expressions to determine significance. A volcano chart analysis showed the positively (right) and negatively (left) correlated genes that were differentially expressed (Figure 4A), and a heatmap indicated the top 10 positively associated genes (Figure 4B). Additionally, to explore crucial pathways related to the biological functions of RBP4, we performed KEGG and GO enrichment analyses. The KEGG analysis indicated that RBP4 was associated with complement and coagulation cascades, metabolic pathways, the biosynthesis of antibiotics, the peroxisome proliferator-activated receptor signaling pathway, and pyruvate metabolism pathways (Figure 4C). The GO analysis also included subtype functional enrichment analyses for GO annotation, biological process (BP), cellular component (CC), and molecular function (MF). The results indicated that for the analysis of BP, RBP4 was related to blood coagulation, negative regulation of endopeptidase activity, fibrinolysis, proteolysis, triglyceride homeostasis, and cholesterol homeostasis. The CC analysis showed that RBP4 was associated with blood microparticles, extracellular exosomes, extracellular spaces, very low-density lipoprotein particles, and the extracellular region. The MF analysis demonstrated that serine-type endopeptidase activity, serine-type endopeptidase inhibitor activity, cholesterol transporter activity, phospholipid binding, receptor binding, glycoprotein binding, and heparin binding were all associated with RBP4's biological functions (Figure 4D). These results suggest that RBP4-related genes were involved in various biological functions and may play essential roles in tumor progression.

### PPI network analysis and hub gene identification

**Figure 5:**
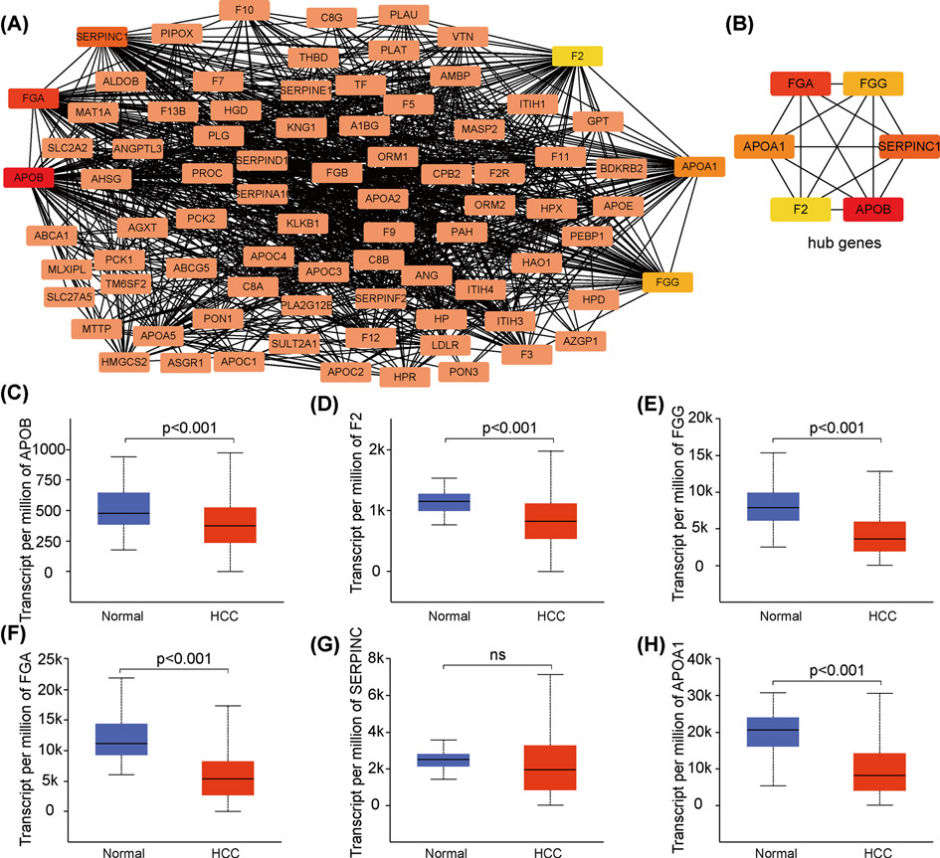
Protein–protein interaction (PPI) network map and hub genes identification (**A**) A representation of the complex PPI network for RBP4 and related proteins. (**B**) The genes *FGA, FG, APOB, F2, SERPINC1*, and *APOA1* were identified as hubs for RBP4 interactions. (**C–H**) The expression levels of *APOB, F2, FGG, FGA*, and *APOAP1* in HCC samples compared with their levels in normal liver samples.

**Figure 6:**
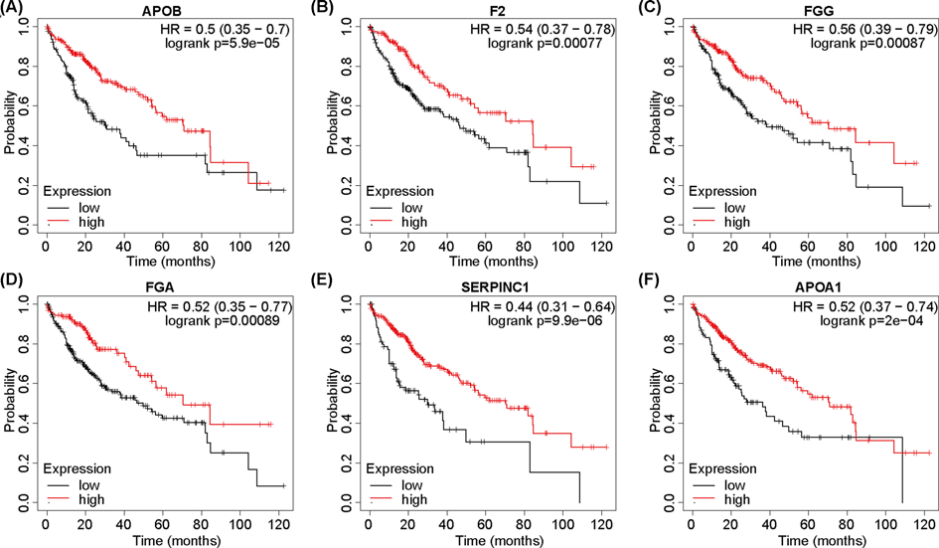
Overall survival rates associated with the expressions of the six hub genes (*FGA, FG, APOB, F2, SERPINC1*, and *APOA1*) (**A–F**) Overall survival rates were significantly correlated with hub gene expression, with high expression levels associated with longer survival time.

To further explore correlations between RBP4 expression and potential hub genes, we constructed a PPI network map for RBP4-related genes (Figure 5A), and then identified the six hub genes *APOB, FGA, FGG, SERPINC1, APOA1*, and *F2* (Figure 5B). Their expression levels are illustrated in Figure 5C–H. The expressions of *APOB, FGA, FGG, APOA1*, and *F2* were significantly down-regulated in the TCGA-LIHC cohort (*P*<0.05), but SERPINC1 levels were not significantly different between normal and tumor tissues (*P*>0.05). Furthermore, we assessed patient OS for the six hub genes, and the high expression levels of *APOB, F2, FGG, FGA, SERPINC1*, and *APOA1* were associated with better prognoses, and coincided with the OS results for RBP4 expression and prognosis (Figure 6A–F).

**Figure 7:**
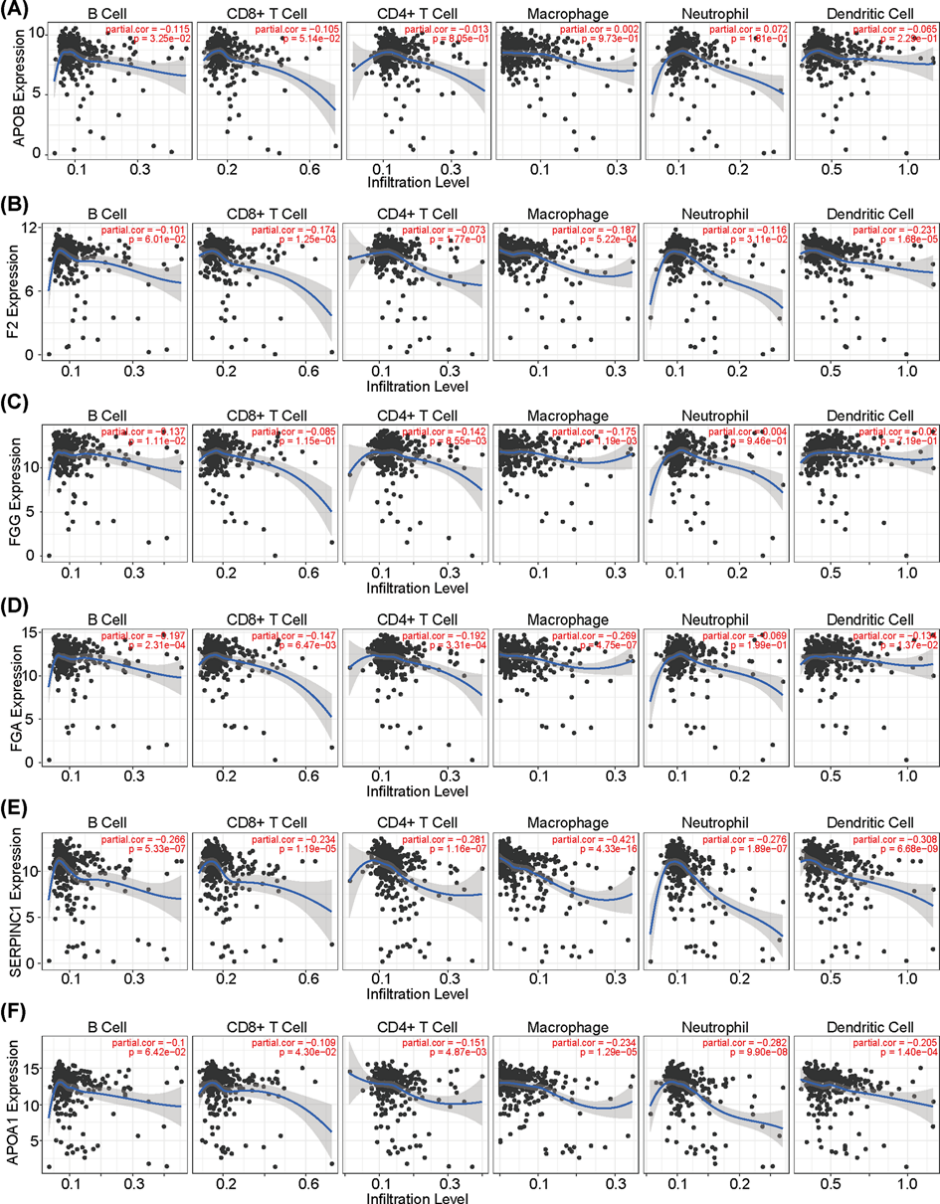
The relationship between hub gene expression and immune cell infiltrates in HCC based on the TIMER dataset (**A**) The relationship between APOBE expression and immune cells infiltration. (**B**) The relationship between F2 expression and immune cells infiltration. (**C**) The relationship between FGG expression and immune cells infiltration. (**D**) The relationship between FGA expression and immune cells infiltration. (**E**) The relationship between SERPINC1 expression and immune cells infiltration. (**F**) The relationship between APOA1 expression and immune cells infiltration.

Immune cell infiltration was also assessed for these six hub genes. Using the TIMER database, we found out that the relationship between the APOBE expression and B-cell infiltration was weak in HCC cohort (partial cor = -0.115, *P*=3.25e-02) and there was no significant difference between APOBE expression and other immune cells infiltration (Figure 7A). According to the infiltration analyses, CD8+ T cells, neutrophils, macrophages, and dendritic cells were negatively associated with F2 expression (Figure 7B), and B cells, CD4+ T cell, and macrophages were also negatively associated with *FGG* expression (Figure 7C). The expression of *FGA* was negatively correlated with the infiltration of B cells, CD8+ T cells, CD4+ T cells, macrophages, and dendritic cells (Figure 7D), and *SERPINC1* expression was significantly correlated with the infiltration of B cells, CD8+ T cells, CD4+ T cells, macrophages, neutrophils, and dendritic cells (Figure 7E). The expression of *APOA1* showed negative correlation with the infiltration of CD8+ T cells, CD4+ T cells, macrophages, neutrophils, and dendritic cells (Figure 7F). These findings suggest that RBP4 related hub genes expression levels were also correlated with immune cells infiltrations.

## Discussion

Many studies, including ours, have shown that RBP4 is an adipocyte protein that is crucially involved in regulating insulin resistance, metabolic syndrome, the inflammation process, and human cancer tumorigenesis [32].

Under normal conditions, RBP4 is mainly secreted in the liver [33]. It is recognized as an adipokine that is associated with metabolic disorders, and in cancers, as a driver for malignant metastasis and tumor angiogenesis in a variety of cancers [17,34]. Here, we determined that RBP4 expression was decreased in HCC cohorts, and acted as a negative regulator for HCC progression [35]. The relationships between RBP4 expression and clinical prognosis, co-expressed genes, hub genes, and the immune system were investigated using bioinformatic analyses. We also determined that the expression level of RBP4 was significantly decreased in HCC tissue compared with that in healthy tissue samples and found that low RBP4 expression was associated with better patient outcomes. The results suggest that RBP4 expression may inhibit tumor progression and serve as an indicator of better prognoses.

We also identified the 10 top co-expressed genes for RBP4 (*RIDA, SLC27A5, HAGH, AZGP1, LDHD, PCK2, GCDH, DCXR, GPT*, and *QDPR*). SLC27A5 has been reported to participate in the transport of fatty acids, in the metabolism of bile acids, and to act as a tumor suppressor in HCC progression [36]. The down-regulation of HAGH expression has been shown to have significant antiproliferative effects on colorectal cancer [37]. AZGP1 has been reported to inhibit colorectal cancer progression via the mTOR signaling pathway and has also been shown to suppress the progression of malignant soft-tissue sarcoma cells [38]. LDHD has been reported to be a regulator of energy metabolism and is involved with tumor cell glycolysis within the tumor microenvironment [39]. In the present study, these RBP4-related genes were also involved in regulating the metabolic microenvironment, indicating that the regulation of cancer cell metabolism can be either direct or indirect via RBP4 expression.

Cells of the immune system are essential regulators for tumor microenvironment homeostasis. We determined that *RBP4* mutations were negatively correlated with immune cell infiltration, indicating that RBP4 expression is closely related to immune cell bioactivity in preclinical and clinical models. RBP4 expression has been reported to be closely related to immune cell infiltration, especially during inflammation [40]. RBP4 can activate macrophages and CD4+ T cells via both TLR4- and JNK-dependent pathways, leading to the production of TNF-α, IL-1β, and IL-6 cytokines [41–43]. RBP4 is likely to be a complex adipokine because its gene expression has also been positively linked to inflammatory factors, CD68 expression, and adipose tissue inflammation [40]. RBP4 has also been determined to activate macrophages and promote proinflammatory cytokines [44] (i.e., TNF-α production via TLR4-dependent pathways). Here, RBP4 expression was negatively correlated with infiltrating macrophages, T cells, B cells, neutrophils, and dendritic cells. Although we have demonstrated that RBP4 is involved in the regulation of proinflammatory cytokines, the underlying mechanisms require further investigation (Table 1).

**Table 1:** Co-expressed genes of RBP4 in HCC based on TCGA cohort

Correlated Gene	Cytoband	Spearman’s correlation	*P*-value
RIDA	8q22.2	0.771	9.57e-70
SLC27A5	19q13.43	0.751	2.19e-64
HAGH	16p13.3	0.735	3.13e-60
AZGP1	7q22.1	0.727	2.23e-58
LDHD	16q23.1	0.723	1.71e-57
PCK2	14q11.2-q12	0.713	2.84e-55
GCDH	19p13.13	0.709	1.79e-54
DCXR	17q25.3	0.708	2.69e-54
GPT	8q24.3	0.704	2.44e-53
QDPR	4p15.32	0.702	5.83e-53

In conclusion, these links between RBP4 and HCC malignancy, metabolic regulation, and immune cell infiltration support the idea of a protective role for RBP4 in HCC, and for its use in predicting HCC outcomes.

## Data Availability

All data were presented in the article.

## Abbreviations

BLCA: bladder urothelial carcinoma
BRCA: breast invasive carcinoma
CHOL: cholangiocarcinoma
HCC: hepatocellular carcinoma
HNSC: head and neck squamous cell carcinoma
KICH: kidney chromophobe
KIRC: kidney renal clear cell carcinoma
LIHC: liver hepatocellular carcinoma
LUAD: lung adenocarcinoma
LUSC: lung squamous cell carcinoma
PRAD: prostate adenocarcinoma
RBP4: retinol-binding protein 4
SKCM: skin cutaneous melanoma
STAD: stomach adenocarcinoma

## References

[1] Bray F., Ferlay J., Soerjomataram I., Siegel R., Torre L. and Jemal A. (2018) Global Cancer Statistics 2018: GLOBOCAN estimates of incidence and mortality worldwide for 36 cancers in 185 countries. CA Cancer J. Clin. 68, 394–424 10.3322/caac.21492 30207593

[2] Liu Z., Jiang Y., Yuan H., Fang Q., Cai N., Suo C. et al. (2019) The trends in incidence of primary liver cancer caused by specific etiologies: results from the Global Burden of Disease Study 2016 and implications for liver cancer prevention. J. Hepatol. 70, 674–683 10.1016/j.jhep.2018.12.001 30543829

[3] Carbone M., Arron S., Beutler B., Bononi A., Cavenee W., Cleaver J. et al. (2020) Tumour predisposition and cancer syndromes as models to study gene-environment interactions. Nat. Rev. Cancer 20, 533–549 10.1038/s41568-020-0265-y 32472073 PMC8104546

[4] Jiang J., Zheng Q., Zhu W., Chen X., Lu H., Chen D. et al. (2020) Alterations in glycolytic/cholesterogenic gene expression in hepatocellular carcinoma. Aging 12, 10300–10316 10.18632/aging.103254 32479426 PMC7346031

[5] Boese A. and Kang S. (2020) Tumor progression of breast cancer during hyperinsulinemic obesity. Trends Mol. Med. 26, 354–356 10.1016/j.molmed.2020.01.014 32277929

[6] Arnold M., Abnet C., Neale R., Vignat J., Giovannucci E., McGlynn K. et al. (2020) Global burden of 5 major types of gastrointestinal cancer. Gastroenterology 159, 335.e15–349.e15 10.1053/j.gastro.2020.02.068 32247694 PMC8630546

[7] Zhang X., Liu Y., Shao H. and Zheng X. (2017) Obesity paradox in lung cancer prognosis: evolving biological insights and clinical implications. J. Thoracic Oncol.: Off. Publ. Int. Assoc. Study of Lung Cancer 12, 1478–1488 10.1016/j.jtho.2017.07.022 28757418

[8] Vidal A. and Freedland S. (2017) Obesity and prostate cancer: a focused update on active surveillance, race, and molecular subtyping. Eur. Urol. 72, 78–83 10.1016/j.eururo.2016.10.011 27771128 PMC5397380

[9] Bailly L., Fabre R., Pradier C. and Iannelli A. (2020) Colorectal cancer risk following bariatric surgery in a nationwide study of French individuals with obesity. JAMA Surg. 10.1001/jamasurg.2020.0089 32159744 PMC7066530

[10] Carreras-Torres R., Johansson M., Gaborieau V., Haycock P., Wade K., Relton C. et al. (2017) The role of obesity, type 2 diabetes, and metabolic factors in pancreatic cancer: a Mendelian Randomization Study. J. Natl. Cancer Inst. 109, 10.1093/jnci/djx012 28954281 PMC5721813

[11] Rajesh Y. and Sarkar D. (2020) Molecular mechanisms regulating obesity-associated hepatocellular carcinoma. Cancers (Basel.) 12, 1290 10.3390/cancers12051290 32443737 PMC7281233

[12] Zabetian-Targhi F., Mahmoudi M., Rezaei N. and Mahmoudi M. (2015) Retinol binding protein 4 in relation to diet, inflammation, immunity, and cardiovascular diseases. Adv. Nutr. (Bethesda, Md.) 6, 748–762 10.3945/an.115.008292 26567199 PMC4642414

[13] Kotnik P., Fischer-Posovszky P. and Wabitsch M. (2011) RBP4: a controversial adipokine. Eur. J. Endocrinol. 165, 703–711 10.1530/EJE-11-0431 21835764

[14] Meex R. and Watt M. (2017) Hepatokines: linking nonalcoholic fatty liver disease and insulin resistance. Nat. Rev. Endocrinol. 13, 509–520 10.1038/nrendo.2017.56 28621339

[15] Fasshauer M. and Blüher M. (2015) Adipokines in health and disease. Trends Pharmacol. Sci. 36, 461–470 10.1016/j.tips.2015.04.014 26022934

[16] Esteve E., Ricart W. and Fernández-Real J. (2009) Adipocytokines and insulin resistance: the possible role of lipocalin-2, retinol binding protein-4, and adiponectin. Diabetes Care 32, S362–S367 10.2337/dc09-S340 19875582 PMC2811453

[17] Papiernik D., Urbaniak A., Kłopotowska D., Nasulewicz-Goldeman A., Ekiert M., Nowak M. et al. (2020) Retinol-binding protein 4 accelerates metastatic spread and increases impairment of blood flow in mouse mammary gland tumors. Cancers (Basel.) 12, 623 10.3390/cancers12030623 32156052 PMC7139568

[18] Jiao C., Cui L., Ma A., Li N. and Si H. (2016) Elevated serum levels of retinol-binding protein 4 are associated with breast cancer risk: a case-control study. PLoS ONE 11, e0167498 10.1371/journal.pone.0167498 28002423 PMC5176270

[19] Wang Y., Wang Y. and Zhang Z. (2018) Adipokine RBP4 drives ovarian cancer cell migration. J. Ovarian Res. 11, 29 10.1186/s13048-018-0397-9 29642915 PMC5896151

[20] Cheng Y., Liu C., Zhang N., Wang S. and Zhang Z. (2014) Proteomics analysis for finding serum markers of ovarian cancer. BioMed Res. Int. 2014, 179040 10.1155/2014/179040 25250314 PMC4164372

[21] Lorkova L., Pospisilova J., Lacheta J., Leahomschi S., Zivny J., Cibula D. et al. (2012) Decreased concentrations of retinol-binding protein 4 in sera of epithelial ovarian cancer patients: a potential biomarker identified by proteomics. Oncol. Rep. 27, 318–324 22020625 10.3892/or.2011.1513

[22] Karunanithi S., Levi L., DeVecchio J., Karagkounis G., Reizes O., Lathia J. et al. (2017) RBP4-STRA6 pathway drives cancer stem cell maintenance and mediates high-fat diet-induced colon carcinogenesis. Stem Cell Rep. 9, 438–450 10.1016/j.stemcr.2017.06.002 28689994 PMC5549806

[23] El-Mesallamy H., Hamdy N., Zaghloul A. and Sallam A. (2012) Serum retinol binding protein-4 and neutrophil gelatinase-associated lipocalin are interrelated in pancreatic cancer patients. Scand. J. Clin. Lab. Invest. 72, 602–607 10.3109/00365513.2012.723135 23020231

[24] Koroknai V., Szász I., Hernandez-Vargas H., Fernandez-Jimenez N., Cuenin C., Herceg Z. et al. (2020) DNA hypermethylation is associated with invasive phenotype of malignant melanoma. Exp. Dermatol. 29, 39–50 10.1111/exd.14047 31602702

[25] Wang D., Zhao Y., Wang L., Ren G., Wang F., Xia Z. et al. (2011) Preoperative serum retinol-binding protein 4 is associated with the prognosis of patients with hepatocellular carcinoma after curative resection. J. Cancer Res. Clin. Oncol. 137, 651–658 10.1007/s00432-010-0927-3 20549233 PMC11828303

[26] Wang D.-D., Zhao Y.-M., Wang L., Ren G., Wang F., Xia Z.-G. et al. (2011) Preoperative serum retinol-binding protein 4 is associated with the prognosis of patients with hepatocellular carcinoma after curative resection. J. Cancer Res. Clin. Oncol. 137, 651–658 10.1007/s00432-010-0927-3 20549233 PMC11828303

[27] Kotnik P., Fischer-Posovszky P. and Wabitsch M. (2011) RBP4: a controversial adipokine. Eur. J. Endocrinol. 165, 703–711 10.1530/EJE-11-0431 21835764

[28] Chandrashekar D., Bashel B., Balasubramanya S., Creighton C., Ponce-Rodriguez I., Chakravarthi B. et al. (2017) UALCAN: a portal for facilitating tumor subgroup gene expression and survival analyses. Neoplasia 19, 649–658 10.1016/j.neo.2017.05.002 28732212 PMC5516091

[29] Kim P., Park A., Han G., Sun H., Jia P. and Zhao Z. (2018) TissGDB: tissue-specific gene database in cancer. Nucleic Acids Res. 46, D1031–D1038 10.1093/nar/gkx850 29036590 PMC5753286

[30] Menyhárt O., Nagy Á. and Győrffy B. (2018) Determining consistent prognostic biomarkers of overall survival and vascular invasion in hepatocellular carcinoma. R. Soc. Open Sci. 5, 181006 10.1098/rsos.181006 30662724 PMC6304123

[31] Vasaikar S., Straub P., Wang J. and Zhang B. (2018) LinkedOmics: analyzing multi-omics data within and across 32 cancer types. Nucleic Acids Res. 46, D956–D963 10.1093/nar/gkx1090 29136207 PMC5753188

[32] Mogler C., König C., Wieland M., Runge A., Besemfelder E., Komljenovic D. et al. (2017) Hepatic stellate cells limit hepatocellular carcinoma progression through the orphan receptor endosialin. EMBO Mol. Med. 9, 741–749 10.15252/emmm.201607222 28373218 PMC5452049

[33] van Dam R. and Hu F. (2007) Lipocalins and insulin resistance: etiological role of retinol-binding protein 4 and lipocalin-2? Clin. Chem. 53, 5–7 10.1373/clinchem.2006.080432 17202496

[34] Komor M., Bosch L., Coupé V., Rausch C., Pham T., Piersma S. et al. (2020) Proteins in stool as biomarkers for non-invasive detection of colorectal adenomas with high risk of progression. J. Pathol. 250, 288–298 10.1002/path.5369 31784980 PMC7065084

[35] Kinoshita M. and Miyata M. (2002) Underexpression of mRNA in human hepatocellular carcinoma focusing on eight loci. Hepatology 36, 433–438 10.1053/jhep.2002.34851 12143053

[36] Gao Q., Zhang G., Zheng Y., Yang Y., Chen C., Xia J. et al. (2020) SLC27A5 deficiency activates NRF2/TXNRD1 pathway by increased lipid peroxidation in HCC. Cell Death Differ. 27, 1086–1104 10.1038/s41418-019-0399-1 31367013 PMC7206086

[37] Ho I., Abdul Aziz A. and Mat Junit S. (2020) Evaluation of anti-proliferative effects of barringtonia racemosa and gallic acid on Caco-2 cells. Sci. Rep. 10, 9987 10.1038/s41598-020-66913-x 32561807 PMC7305318

[38] Yu W., Ling J., Yu H., Du J. and Liu T. (2020) AZGP1 suppresses the process of colorectal cancer after upregulating FASN expression via mTOR signal pathway. Gen. Physiol. Biophys. 39, 239–248 10.4149/gpb_2019061 32525817

[39] Riddell S., Weinerman B., Kemel S., Schipper H. and Vadas G. (1980) The treatment resistance of lymphocyte depleted Hodgkin’s disease. Cancer 46, 1503–1508 10.1002/1097-0142(19800915)46:6<1503::AID-CNCR2820460635>3.0.CO;2-0 7417952

[40] Yao-Borengasser A., Varma V., Bodles A., Rasouli N., Phanavanh B., Lee M. et al. (2007) Retinol binding protein 4 expression in humans: relationship to insulin resistance, inflammation, and response to pioglitazone. J. Clin. Endocrinol. Metab. 92, 2590–2597 10.1210/jc.2006-0816 17595259 PMC2893415

[41] Lee S., Yuen J., Jiang H., Kahn B. and Blaner W. (2016) Adipocyte-specific overexpression of retinol-binding protein 4 causes hepatic steatosis in mice. Hepatology 64, 1534–1546 10.1002/hep.28659 27227735 PMC5074895

[42] Moraes-Vieira P., Yore M., Dwyer P., Syed I., Aryal P. and Kahn B. (2014) RBP4 activates antigen-presenting cells, leading to adipose tissue inflammation and systemic insulin resistance. Cell Metab. 19, 512–526 10.1016/j.cmet.2014.01.018 24606904 PMC4078000

[43] Viard H., Mabille J., Favre J., David M., Grevy A. and Sgro J. (1974) Splenic artery aneurysm. Apropos of 3 cases. Ann. Chir. 28, 183–188 4839203

[44] Norseen J., Hosooka T., Hammarstedt A., Yore M., Kant S., Aryal P. et al. (2012) Retinol-binding protein 4 inhibits insulin signaling in adipocytes by inducing proinflammatory cytokines in macrophages through a c-Jun N-terminal kinase- and toll-like receptor 4-dependent and retinol-independent mechanism. Mol. Cell. Biol. 32, 2010–2019 10.1128/MCB.06193-11 22431523 PMC3347417

